# Investigating suicidal behavior among prisoners in the light of the behavioral addiction approach: results of a multicenter cross-sectional study

**DOI:** 10.3389/fpsyt.2024.1448711

**Published:** 2024-07-25

**Authors:** Irina Horváthné Pató, Szilvia Kresznerits, Tamás Szekeres, Ágnes Zinner-Gérecz, Dóra Perczel-Forintos

**Affiliations:** ^1^ National Prison, Psychological Department, Szombathely, Hungary; ^2^ Mental Health Sciences Division, Doctoral School of Semmelweis University, Budapest, Hungary; ^3^ Department of Clinical Psychology, Faculty of Medicine, Semmelweis University, Budapest, Hungary; ^4^ Department of Internal Medicine and Oncology, Semmelweis University, Budapest, Hungary

**Keywords:** inmates, suicide, self-harm, addictive behavior, depression, impulsivity, emotion regulation, mindfulness

## Abstract

**Introduction:**

The behavioral addiction model posits that repetitive suicidal behaviors can serve as maladaptive strategies for managing stress and negative emotional states, akin to substance addiction. Both behaviors involve negative emotions, offer temporary psychological relief, and persist, indicating shared neurobiological mechanisms. This study explored psychometric differences among major repeaters, occasional attempters, and non-suicidal prisoners.

**Methods:**

A multi-centre cross-sectional survey of 363 inmates across four prisons assessed depression, cognitive-emotional regulation, impulsivity, perceived stress, lifetime non-suicidal self-injury and suicide attempts.

**Results:**

Mild depression, moderate suicidal ideation, and moderate impulsivity were common, with nearly half of the participants having attempted suicide at least once. Hierarchical multiple regression analyses revealed that repeated suicidal behavior in the past increases susceptibility to future suicidal thoughts, with suicide attempts serving as a maladaptive emotion regulation strategy among repeated attempters.

**Discussion:**

The results reveal differences in emotional dysregulation, impulsivity, and stress coping strategies among the studied groups, reinforcing the idea of suicidality as a form of behavioral addiction. The addiction approach helps explain the sensitivity to later suicidal thoughts in former attempters and self-harmers, offering valuable insights for tailored interventions within correctional settings.

## 1 Introduction

In several countries suicidality in prison is the leading cause of death (1–5). This phenomenon is not necessarily linked to incarceration. In many countries, including Hungary, the number of completed suicides and attempted suicides during incarceration is extremely low (6–8). However, the incarcerated population is significantly more at risk of suicide during their entire life history: among prisoners 8.6% of men (95% CI 6.1-11.2) and 12.2% of women (95% CI 7.1-17.3) attempt suicide over the course of a lifetime, compared with 2.7% in general population (9). It could mean that prisoners belonged to a risk group even before incarceration, and that the risk of suicide attempts also can be increased even after release. This disparity highlights the crucial need to understand the factors contributing to the high rates of suicide attempts, thereby facilitating the establishment of appropriate screening procedures and interventions.

Meta-analyses (2, 4) have identified key risk factors associated with suicide attempts and self-harm among incarcerated individuals, including factors preceding suicide, comorbid mental disorders, single-person cell placement, lack of visits, abuse during incarceration, violent offenses, life sentences, and remand. The strongest clinical factors associated with an increased risk of suicide include current or recent suicidal ideation, which raises the likelihood of suicide by over ten times. Additionally, a history of previous non-suicidal self-injury (NSSI) or suicide attempts (SA) represents an eight-fold risk (2, 4).

Prisoners are exposed to heightened levels of chronic stress, stemming from interpersonal, financial, and legal factors. The emotionally taxing nature of prison life necessitates effective coping mechanisms, often leading to the development of adjustment and mental disorders (10, 11). The chronic stress factors affecting prisoners often precede the time of imprisonment, they are often exposed to childhood and adult traumas (abandonment, emotional, physical or sexual traumas as well) (12). During incarceration, prisoners face stressors such as the possible disruption of partner and family relationships, lack of social support, being assaulted; having major financial difficulties or institutional conflicts with other inmates or staff (10). The primary concerns can manifest as substance-related and addictive disorders, impulsivity-related conditions such as personality disorders, impulse control disorders, and major depression (13–18). Despite the elevated incidence of mental disorders among incarcerated individuals, coupled with their heightened vulnerability, healthcare resources in this context remain limited (13).

Several theories and research have investigated the risk factors of suicide in the general population. One of the most comprehensive models for suicide attempts is the integrated motivational-volitional (IMV) model of suicidal behavior by O’Connor and Kirtley (19). The model underscores vulnerability to suicide across three phases: pre-motivation, motivation, and action. The pre-motivation phase considers diathesis, environmental factors, and early life events that underlie suicidal ideation. The motivation phase centers on entrapment due to failure and humiliation, influenced by variables affecting self-regulation. The action phase involves transitioning from thought to suicide attempt, influenced by access to means, pain sensitivity, and impulsivity (19).

Additionally, various models delve into the complexities of NSSI. The cognitive-emotional model (20) posits that emotional reactivity, mental representation of self-harm, negative self-representations, and thoughts related to NSSI contribute to it as a maladaptive coping strategy. Similarly, the four-function model (21) describes processes maintaining NSSI through automatic/social and negative/positive reinforcement, primarily serving intrapersonal functions, such as emotion regulation and reducing negative emotional states. Furthermore, the cognitive-emotional reactivity model (22) integrates insights from recurrent depression, applying them to borderline personality disorder, where maladaptive coping strategies like self-harm reinforce connections between negative thoughts, emotions, and bodily sensations. Therefore, NSSI becomes more and more automatic because of the repetitions.

Research has investigated the potential similarities between SA, NSSI, and behavioral addictions (23–28). This concept challenges traditional views and suggests that considering recurrent suicidal behavior as an addiction could transform treatment approaches, perhaps comparing them with approaches typically used for substance addiction.

Behavioral addictions are triggered by specific patterns and involve compulsive engagement in a particular activity, despite negative consequences. It should be mentioned that these addictions are not officially recognized in the DSM-5. Unlike substance addictions, behavioral addictions are defined by behaviors that become overwhelmingly dominant in an individual’s life, profoundly impacting their daily functioning, physical and mental health, and the surrounding environment (29). While this classification remains a subject of ongoing debate among experts, numerous studies have unearthed intriguing connections that warrant exploration.

The addictive model of self-harming behavior by Blasco-Fontecilla, Fernández-Fernández (25) emphasizes that individuals with frequent SA or NSSI (≥5 attempts in a lifetime) may be considered behavioral addicts. The recurrence of self-harm and SA is primarily linked to negative emotions, such as relieving emptiness or helplessness seeking attention. This model suggests common neurobiological mechanisms, including the involvement of the mesocorticolimbic reward system and the release of endogenous opioids, shared between self-harm and addiction. Both self-harm and addiction provide relief from psychological pain and activate the stress and opioid systems. Similarly to addiction, self-harming behaviors may become more persistent and severe over time. Self-harm can sensitize individuals to suicidal thoughts and behaviors, making them more susceptible to triggering by stressful life events. There is also a risk of relapse in both self-harm and addiction, often precipitated by similar life events (25).

The urge to self-harm in individuals with NSSI parallels addictive features (30), including increased repetition, impulsivity, rumination, and withdrawal symptoms (24, 25, 31). Higher scores at the addictive features subscale of the Ottawa Self-Injury Inventory are associated with greater distress over urges to self-injure, and also with more frequent and severe NSSI (28). Analyzing the language used in forum posts related to NSSI, Himelein-Wachowiak, Giorgi (27) found that over 75% of the users in their sample fulfilled a minimum of two NSSI-adapted diagnostic criteria of substance use disorders in their posts.

Adolescent studies on NSSI also found addictive features, displaying an underlying urge, increasing severity and frequency, difficulty in stopping self-harming behavior, and relief after self-harm (26). High impulsivity and emotional dysregulation were identified as major risk factors for self-harm, leading to the characterization of certain elements of self-harm as behavioral addiction (32). Childhood abuses and traumas were frequently observed among adolescents with NSSI, and three out of five psychiatric outpatients exhibited addictive features in their self-harming behavior, often co-occurring with other substance or behavioral addictions (33).

Our study examines the connection between traditional addiction concepts and the understanding of suicidal behavior as an addiction. It explores the implications of this connection specifically within the incarcerated population.

## 2 Measures and methods

### 2.1 Procedures and sample

Our cross-sectional multi-center survey was conducted between June-August of 2021, included prisoners (N=363) from 4 prisons on a voluntary basis. Based on sample size calculation (34) a total of 232 participants were required, considering a 95% confidence interval with a 5% margin of error. The study was carried out persons convicted by final judgement. Exclusion criteria were psychological condition preventing the completion of the test, such as acute psychosis, severe intellectual disability, and psychoactive substance abuse, based on previous medical records or on the advices of the institutional psychologist or psychiatrist.

A psychologist remained present throughout the test session to offer assistance with question interpretation.

### 2.2 Measures

Participants completed questionnaires below in the following order:

*Individual data sheet:* Following the informed information and consent section, participants provided demographic information. This included gender, age, education, employment, marital status, and, for incarcerated individuals, the reason for and duration of detention.

*NSSI occurrence questions*: “Have you ever intentionally harmed yourself with suicide intention?” [yes/no]. If the respondent answers “Yes”, further questions were asked about the frequency (“If yes, how many times?” [1/2/…/6/more than 6]) and the way (“How did you do it?”) of self-harm.

*Cognitive emotion regulation questionnaire (CERQ)*: a 36-item self-report questionnaire in which the respondent determines how he or she reacts to negative, unpleasant events on a five-point Likert scale (1-5 points) (35). The nine subscales (36, 37), each with four items, examine five adaptive (acceptance, positive focus, planning, positive reappraisal, perspective taking) and four non-adaptive (self-blame, rumination, catastrophizing, blaming others) cognitive emotion regulation mechanisms.

*Five facet mindfulness questionnaire (FFMQ):* 39-item self-report questionnaire designed to measure mindfulness as a personality trait, the five subscales are observation, description, act with awareness, non-judgment, and non-reactivity (38). We used the scale as one-dimensional.

*Beck depression inventory (BDI-S)*: a 9-item self-report scale measuring the severity of depression on a 4-point Likert scale (0-3 point) from ‘not at all’ to ‘completely agree’. The most reliable cut-off point was found to be 10 points; however, in the case of depression independent of anxiety, the questionnaire already indicates a clinical level from 6 points (39, 40).

*Perceived stress scale (PSS4)*: a 4-question, 5-point Likert scale (0-4 point) asking about thoughts and feelings that characterize a person’s perception of stress (41, 42).

*Short version of the Barratt Impulsivity Scale (BIS-S-8)*: an 8-item self-report questionnaire in which the respondent indicates on a four-point Likert scale (1-4 point) how often he or she is likely to make the statement (43, 44).

*Paykel suicide scale (PAYKEL)*: a 5-item self-report questionnaire designed to explore the respondent’s suicidal thoughts, ideation and attempts in the past two weeks on a 4-point Likert scale (0-4 point) (45). *Questions on SA*: In addition, separate questions assess whether the respondent has had a suicide attempt in the past, with a yes/no responses. If the respondent answered “Yes” to the last question of the Paykel Suicide Scale, two additional questions were added to the questionnaire: on the frequency of suicide attempts (“If yes, how many times?” [1/2/…/6/more than 6]) and on the method (“How did you make the attempt?”).

### 2.3 Ethics

All subjects were informed about the study, and all provided informed consent. All data were anonymized before processing, and participants had the right to withdraw their data from the research at any time without providing a reason in accordance with the Ethical Code for Psychologists adopted by the Hungarian Psychological Association and the Hungarian Psychological Society. The research was approved by regional and institutional ethical committees.

### 2.4 Data analysis

Data analysis was conducted with IBM SPSS Statistics 28^©^ and JASP for hierarchical multiple regression analysis. Missing values were excluded from the analysis. A significance level (α) of.05 or less was considered significant. Cronbach’s alpha analysis was conducted for reliability assessment of the measurement tools used in the study (**Table 1**).

**Table 1:** Descriptive statistics of the psychological questionnaires with Cronbach’s α value of reliability analysis.

Variables	Mean (SD)	Cronbach’s α
BDI-S	6.011 (5.458)	.842
PAYKEL	2.154 (4.087)	.916
FFMQ	127.265 (17.698)	.858
BIS-S-8	16.398 (4.609)	.746
CERQ adaptive	64.052 (15.706)	.896
CERQ non-adaptive	44.154 (12.516)	.856
PSS4	6.647 (3.243)	.702

BDI-S, Beck Depression Inventory Shortened; PAYKEL, Paykel Suicide Scale; FFMQ, Five Facet Mindfulness Questionnaire; BIS-S-8, Barratt Impulsivity Scale Shortened; CERQ, Cognitive Emotion Regulation Questionnaire; PSS4, Perceived Stress Questionnaire Shortened.

As the hypothesis of a normal distribution was rejected (Shapiro-Wilk test), the frequencies of SA and NSSI were categorized as follows. Inmates were classified into four subgroups based on SA and NSSI: 1) major repeater (MR) - total lifetime SA or NSSI ≥ 5, 2) repeated attempter (RA) - total SA or NSSI between 2-4 times, 3) one-time attempter (OA), and 4) non-attempter (NA). Binary logistic regression models with Wald forward method were employed to examine potential differences in psychological characteristics (level of depression, cognitive-emotional strategies, impulsivity, mindfulness skills and perceived stress) among these groups concerning suicidal behavior (MR, RA, OA, or NA in the case of NSSI and SA). There is no collinearity or multi-collinearity between the variables, and we have checked for collinearity prior to the analysis of the binary logistic regression (r<0.62 and VIF=1.068-1.929 in all cases).

Hierarchical multiple regression analyses were conducted to examine four models: the moderating effects of previous NSSI and SA on the relationship between perceived stress and suicide thoughts, and between depression level and suicide thoughts.

## 3 Results

### 3.1 Reliability indexes and mean scores

The baseline characteristics of the sample are shown in **Table 2**.

**Table 2:** Baseline characteristics of the inmate sample (N = 363).

Variable	Frequency / Mean (SD)
Age	39.54 years (range 21-75 ys, SD = 10.07)
Sex (female : male)	48.2% (N= 175) : 51.8% (N = 188)
Education	11.3% lower than basic education 40.5% primary education 43.3% secondary education 5.0% higher education
Marital status	33.1% single 30.0% stable relationship 17.9% married 14.9% divorced 4.1% widowed
Reason of incarceration	46.7% non-violent 53.2% violent
Average time spent in prison during lifetime	84.46 months (SD = 60.66 months)
Time spent in prison during lifetime	9.5% less than a year 12.1% 1-2 years 31.2% 2-5 years 29.0% 5-10 years 18.2% more than 10 years

**Table 1** shows the reliability indexes and mean scores of scales. All of the baseline Cronbach’s alpha scores were appropriate for early stage studies (α>.70) based on recommendation (46). Prisoners have on average mild level of depression, moderate level of suicide thoughts, moderate impulsivity and perceived stress level, with normal adaptive and maladaptive cognitive emotional strategies.

### 3.2 Characteristics of suicidal behavior

In terms of suicidal behavior, the sample distribution was as follows: 38.4% (N=101) of prisoners had a history of lifetime NSSI. Among them, 38.4% engaged in NSSI once, 35.6% were repeated attempters (RA) with 2-4 attempts, and 31.7% were major repeaters (MR) with at least 5 NSSI incidents during their lifetime. The most common method of NSSI was cutting (77.2%), followed by hitting themselves till tissue harm (6.9%) and burning themselves (5.9%).

Additionally, 43.3% (N=114) of inmates had attempted suicide previously, with 41.3% being once attempters (OA), 33.3% RA, and 25.4% MR. The most frequent suicide methods were via drugs or self-poisoning (42.1%), cutting (39.5%), and self-hanging (20.2%).

We conducted exploratory binary logistic regression models including all significant psychometric variables such as level of depression, suicide thoughts, mindfulness, emotion regulation skills, impulsivity and perceived stress in relation to the frequency of SA or NSSI (**Tables 3**, **4**). Out of the six models, two were found to be significant with acceptable explanatory power (Nagelkerke R^2^ > 0.2). These models examined the characteristics associated with SA (Model 2 and 3).

**Table 3:** Model coefficients and summary indicators of binary logistic regression models.

	χ^2^	df	p	-2 Log likelihood	Cox & Snell R^2^	Nagelkerke R^2^
Model 1: SA_MR	16.996	2	<.001	158.759	.056	.125
Model 2: SA_RA	46.130	2	<.001	248.660	.145	.229
Model 3: SA_A	74.500	3	<.001	296.936	.224	.312
Model 4: NSSI_MR	9.378	2	.009	175.543	.031	.067
Model 5: NSSI_RA	20.768	3	<.001	276.765	.068	.107
Model 6: NSSI_A	14.097	3	.003	341.284	.047	.067

SA, suicide attempts; NSSI, non-suicidal self-injury; MR, multirepeater attempt as reference category; RA, repeated attempt as reference category; A, attempt of suicide or NSSI in lifehistory as reference category. Method, Wald backward.

**Table 4:** Statistical indicators of variables in the equation of binary logistic regression models.

	Wald χ^2^	p	OR	95% CI
Model 1: SA_MR				
CERQ non-adaptive	9.789	.002	.940	.905-.977
BIS-S-8	4.896	.027	1.106	1.012-1.210
Model 2: SA_RA				
CERQ_non-adaptive	21.608	<.001	.933	.905-.960
PAYKEL	18.122	<.001	1.141	1.074-1.213
Model 3: SA_A				
CERQ adaptive	3.426	.064	1.017	.999-1.035
CERQ non-adaptive	20.388	<.001	.944	.921-.968
PAYKEL	34.679	<.001	1.243	1.156-1.337
Model 4: NSSI_MR				
PAYKEL	9.509	.002	1.137	1.048-1.233
FFMQ	3.796	.051	1.025	1.000-1.050
Model 5: NSSI_RA				
PSS4	3.093	.079	1.105	.989-1.234
PAYKEL	9.134	.003	1.121	1.041-1.208
FFMQ	7.750	.005	1.029	1.008-1.049
Model 6: NSSI_A				
PSS4	5.412	.020	1.124	1.019-1.241
PAYKEL	2.913	.088	1.061	.991-1.135
FFMQ	3.119	.077	1.016	.998-1.033

SA, suicide attempts; NSSI, non-suicidal self-injury; MR, multirepeater attempt as reference category; RA, repeated attempt as reference category; A, attempt of suicide or NSSI in lifehistory as reference category; CERQ, Cognitive Emotional Regulation Questionnaire; BIS-S-8, Barratt Impulsivity Scale Shortened; PAYKEL, Paykel Suicide Scale; FFMQ, Five Facet Mindfulness Questionnaire; PSS4, Perceived Stress Scale; Method: Wald backward.

Individuals who engaged in repeated SA repeatedly during lifetime, exhibited lower level of non-adaptive emotional regulation strategies and elevated level of suicidal ideation compared to NA or OA individuals (**Table 4**). A similar, yet more pronounced, pattern was observed in the model exploring the characteristics of those with a history of SA. Those who attempted suicide during their life history can be characterized by more intense suicidal thoughts even in the present, and their non-adaptive strategies were lower. Notably, the effect of adaptive strategies was not found to be significant in the model (**Table 4**).

None of the models explaining previous NSSI demonstrated adequate explanatory power (Nagelkerke R^2^ <0.2) (**Table 3**). Therefore, the observed effects can be considered as potential tendencies that require further investigation. The presence of a higher level of suicidal thoughts, elevated perceived stress, and surprisingly, a higher level of mindfulness skills, appeared to be outlined as tendencies among those who had previously self-injured compared to those who never committed NSSI (**Table 4**).

### 3.3 Hierarchical regression analysis: the moderator role of previous suicide behavior to the relationship of depression and suicide ideation

Using hierarchical regression (HR), we analyzed the extent to which the number of suicidal ideations (PAYKEL) is predicted by the level of depression (BDI-S) or perceived stress (PSS4), and how previous NSSI or SA moderates this relationship. No one-dimensional outlier excluding the trial was found in the data based on the outlier labelling rule (Tukey, 1977).

**Figure 1:**
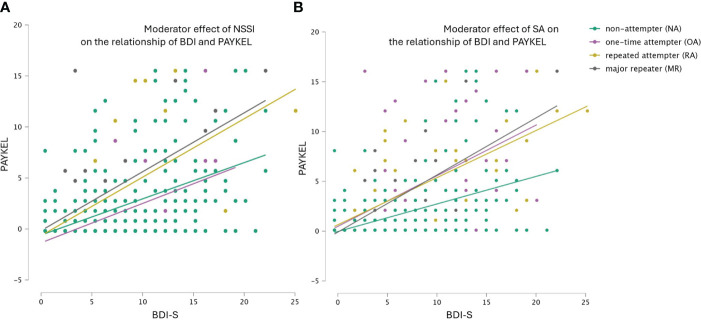
**(A, B)** Hierarchical regression model 1: Moderator effect of previous non-suicidal self-injury (NSSI) and previous suicide attempts (SA) on the association of depression (BDI-S) with suicidal ideations (PAYKEL).

HR model 1 (**Figure 1**): In the first model we examined the relationship between depression and suicide thoughts (BDI-S ➔ PAYKEL). The initial model was significant (F(2.360) = 82.207 p<.001). Depression explained 31% of the variance in suicidal ideation (R^2^Adj = .314). At second level we entered NSSI as a moderator into the model (HR model 1a), which had a significant moderator effect (p<.001). Including the interaction in the model the explained variance increased significantly by 1.2% (F(1.359) = 6.581, p<.05). When examining the interaction of depression on suicidal ideation (BDI-S ➔ PAYKEL) with SA (HR model 1b), the moderating effect of previous attempts of suicide was also significant (p<.001) and obtained results were consistent with the previous moderation: including SA in the model increased the explained variance significantly (F(1.359) = 8.627, p<.05) by 1.6%.

**Figure 2:**
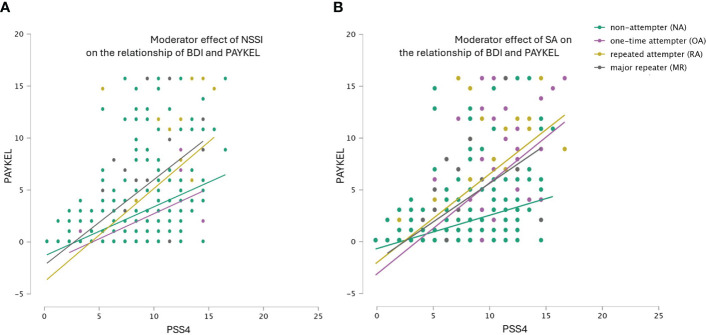
**(A, B)** Hierarchical regression model 2: Moderator effect of previous non-suicidal self-injury (NSSI) and previous suicide attempts (SA) on the association of perceived stress (PSS4) with suicidal ideations (PAYKEL).

HR model 2 (**Figure 2**): In the second model, we examined the relationship between perceived stress and suicide thoughs (PSS4 ➔ PAYKEL), the model was significant (F(2.360) = 76.326 p<.001). Perceived stress explained 31% of the variance in suicide (R^2^Adj = .317. The effects of the interactions (HR model 2a: NSSI x PSS4 ➔ PAYKEL and HR model 2b: NSSI x PSS4 ➔ PAYKEL) were also significant (p<.001). Including the moderating variables in the model increased the explained variance for both previous SA and NSSI by 2.4%, the increases were significant in both cases [F(1.359) = 12.83, p<.001].

## 4 Discussion

Suicidality in prison is a major issue, with incarcerated individuals showing higher lifetime suicide attempt rates compared to the general population (9). This study explores the connection between suicidality and behavioral addictions, highlighting that recurrent suicidal behavior may function similarly to addiction. Both behaviors involve negative emotions, provide psychological relief, and can become more persistent over time (23–28). This addiction-like nature of suicidality indicates shared neurobiological mechanisms (25) and highlights the need for targeted interventions within the incarcerated population.

Our multicentre cross-sectional study involved 363 incarcerated individuals. At the study’s outset, a standardized Hungarian questionnaire for assessing suicide or NSSI as behavioral addictions was unavailable, and questions regarding addiction posed challenges during the correctional institutions’ approval process. Therefore, we assessed the behavioral addiction approach indirectly. We deliberately chose measurement tools—assessing depression, cognitive-emotional regulation, impulsivity, mindfulness skills, perceived stress, and lifetime incidents of self-harm and suicide attempts—to capture multifaceted aspects of suicidal behavior among inmates. These instruments are suitable for assessing both suicidal behavior and the underlying psychological mechanisms of behavioral addiction. The datasheet on SA and NSSI helped assess the frequency and severity of suicidal behaviors. Although inmates could provide open-ended responses for triggers, this method proved unreliable, and the data were not analyzed. A semi-structured interview would likely be more effective. Negative emotions such as emptiness or helplessness and elevated stress levels are primary triggers for both self-harm and addiction (19, 22, 25). To assess these, we administered the BDI-S and PSS4. The PAYKEL scale provides information on suicidal thoughts, plans, and intentions, central to the second phase of the IMV model (19) in understanding suicidal behavior. From the perspective of behavioral addictions, it offers insights into craving. High impulsivity and emotional dysregulation increase the risk of self-harm, sharing addictive features such as repetition, difficulty stopping, and relief after self-harm. These factors contribute to the persistence and severity of suicidal behaviors, like substance addiction, especially in individuals with a history of trauma or abuse (30–33). These factors relate to the third phase of the IMV model, turning ideas into action (19). To assess these factors, we used the BIS-S-8, CERQ, and FFMQ.

The analysis revealed the prevalence of mild depression, moderate suicidal ideation, and impulsivity, coupled with a notable history of suicide attempts and self-harm among our sample, underscores the urgent need for tailored interventions. Our findings align with the addiction model, which posits that frequent engagement in suicidal behaviors sensitizes individuals to heightened suicidal ideation during stressful periods. This sensitization mirrors the craving concept in substance addictions, highlighting a potential neurobiological basis shared between behavioral and substance dependencies. We observed a prevalence of mild depression, moderate suicide thoughts, moderate impulsivity, and slightly increased perceived stress in the sample. Furthermore, particularly high percentage of the inmates in our sample attempted suicide or committed non-suicidal self-harm during their life history. Nearly half (43.3%) of the participants attempted suicide at least once. This rate is significantly higher than the approximately 10% reported in large sample studies (9), and also it represents an increased risk for suicidal behavior based on the motivation phase of the IMV model of suicidal behavior (19). According to Hungarian data, this phase usually does not turn into the action phase during detention. Based on 2020 data, the completed suicide rate is 0.36 per 100.000 inmates in Hungarian prisons (8), which is among the lowest rates globally (47). Based on the IMV model, this indicates that the system sets a suitable limit for access to the means and that suicide attempts cannot take place under controlled conditions. However, after release, the possibility of access to means increases, as well as impulsive behavior would be controlled less, so the likelihood of suicidal behavior may increase also. The Hungarian example confirms that restrictive actions and control are extremely important in reducing suicidal behavior during detention, however the deeper understanding of motivational factors can also help to reduce the occurrence of later suicidal behavior.

We hypothesized that major repetition (at least 5 times during life history) of suicide attempts (SA) or non-suicidal self-injury (NSSI) would represent a distinct suicidal among repeaters. Our binary logistic analysis did not confirm this hypothesis, but certain trends were identified (**Tables 3**, **4**). A more frequent history of SA in the past (Model 1-3) correlated with a heightened manifestation of suicidal ideation in the present. This result is consistent with the addiction approach, which suggests that more frequent suicidal behavior sensitizes individuals to later suicidal thoughts in stressful life events (25). The elevated level of suicidal ideation involved not only a cognitive aspect but also an urge and planning, as defined by the Paykel Suicidal Thoughts Scale. This characteristic is comparable to cravings in addictions (24–26, 28, 31). Additionally, individuals engaging in repeated SA exhibited lower levels of non-adaptive emotion regulation strategies (Model 2). Suggesting that suicidal behavior may serve an emotion regulation function, particularly accentuated in those with more frequent attempts. Suicide behavior, in this context, may be distinguished from cognitive emotion regulation strategies, potentially taking precedence as a maladaptive coping mechanism.

Regarding NSSI (Model 4-6), similar tendencies in suicidal thinking were observed, but without noticeable differences in emotion regulation strategies. Conversely, perceived stress levels increased, both in the relationship between self-injurious and non-self-injurious individuals and with an escalation in the number of self-injuries in their life history. This could suggest a diminished level of frustration tolerance in those reporting more frequent self-harm. It is crucial to emphasize that these differences are subtle and indicative of trends, warranting further investigation.

Hierarchical regression models (**Figures 1**, **2**) supported the addictive model of suicidal behavior. At comparable levels of perceived stress or depression, inmates with a history of more frequent NSSI or SA reported a higher frequency of suicidal thoughts than those with less frequent suicidal behavior throughout their life history. Low distress tolerance or frustration tolerance are also key factors of substance or behavioral addiction. The difficulty in sustaining negative emotions plays an important role not only in the development of addictive disorders but also in their maintenance and relapses (48, 49). This finding aligns also with the sensitization theory of the addiction approach, suggesting that repeated suicidal behavior may affect neurobiological mechanisms related to stress and relief systems (25).

After release the risk of suicide among former inmates can be seven times higher than in general population (50), that is why it is very important to identify the factors that may be risk factors for later suicide, even during detention. Understanding this aspect is particularly crucial for prisoners, as incarceration often limits access to means and opportunities for suicide, despite the elevated stress levels they may experience (10), inmates are under controlled conditions. However, upon release, this controlled environment dissolves, significantly amplifying the risk of suicide, especially for those who previously relied on this kind of maladaptive emotion regulation patterns. Based on our results, severe repeater inmates are at a higher risk of future suicide attempts. They exhibit a behavioral pattern that resembles addiction, suggesting that their suicidal behavior could be interpreted as a form of dependence. In their cases even minor stressors or depressive episodes can substantially escalate suicidal ideation during the post-release period. The addiction perspective can assist in their treatment, with a focus on addressing specific behavioral and cognitive patterns.

### 4.1 Limitations

An inherent limitation of our study lies in its cross-sectional design and reliance on retrospective data regarding past instances of self-harm and suicide attempts, which may introduce biases and distortions. Unfortunately, in this study, we were unable to distinguish between the frequency and methods of suicidal behavior occurring before incarceration and those developing since the start of incarceration. However, if future approval processes allow for this distinction, it would be an important aspect in understanding the background of suicidal behavior in this population.

Another limitation is that a questionnaire directly measuring behavioral addiction was not administered. However, based on the open-ended responses on the datasheet, the quantitative methodology also presents limitations in understanding certain relationships within the prison environment. Given the sensitivity of the subject matter, employing qualitative methods could offer deeper insights into suicidal behavior among prisoners. Nonetheless, the study’s multicentric nature and its focus on understanding the suicidal tendencies of a hard-to-reach population contribute to its strength, alongside its relatively large sample size.

### 4.2 Conclusion

In conclusion, our research examines the factors of the motivational phase of suicidal behavior from the perspective of behavioral addictions. Based on the Hungarian data, it can be seen that institutional-level procedures contribute to keeping suicidal behavior at a low-level during detention. However, the high level of suicidal thoughts, moderate impulsivity and frequency of suicide attempts occurring in the life history in the study sample indicate that it would be advisable to pay attention to the risk factors of the motivational phase during detention to prevent suicide after release. This kind of factors could be improving emotion and impulse regulation or increasing frustration tolerance to decrease the urge to suicidal behaviors in negative emotional states. These findings underscore the necessity of continued research to better comprehend and address the unique challenges faced by incarcerated individuals.

## Data availability statement

The raw data supporting the conclusions of this article will be made available by the authors, without undue reservation.

## Ethics statement

The studies involving humans were approved by Research Ethics Committee of Semmelweis University (TUKEB number: 92/2015) Directorate of the Hungarian Prison Service Headquarters. The studies were conducted in accordance with the local legislation and institutional requirements. The participants provided their written informed consent to participate in this study.

## Author contributions

IHP: Conceptualization, Data curation, Investigation, Project administration, Validation, Writing – original draft. SzK: Conceptualization, Data curation, Formal analysis, Investigation, Methodology, Validation, Visualization, Writing – original draft, Writing – review & editing. TSz: Data curation, Formal analysis, Methodology, Validation, Visualization, Writing – original draft. ÁZ-G: Conceptualization, Validation, Writing – original draft. DP-F: Conceptualization, Funding acquisition, Project administration, Resources, Supervision, Validation, Writing – review & editing.

## References

[1] Favril L Indig D Gear C Wilhelm K. Mental disorders and risk of suicide attempt in prisoners. Soc Psychiatry Psychiatr Epidemiol. (2020) 55:1145–55. doi: 10.1007/s00127-020-01851-7 32144468

[2] Favril L Yu R Hawton K Fazel S. Risk factors for self-harm in prison: A systematic review and meta-analysis. Lancet Psychiatry. (2020) 7:682–91. doi: 10.1016/S2215-0366(20)30190-5 PMC7606912 32711709

[3] Habtamu E Desalegn D. Suicidal behavior and associated factors among prisoners in dilla town, dilla, Ethiopia 2020: an institutional based cross-sectional study. PloS One. (2022) 17:15. doi: 10.1371/journal.pone.0267721 PMC9094534 35544553

[4] Zhong S Senior M Yu R Perry A Hawton K Shaw J. Risk factors for suicide in prisons: A systematic review and meta-analysis. Lancet Public Health. (2021) 6:e164–e74. doi: 10.1016/S2468-2667(20)30233-4 PMC7907684 33577780

[5] Khezri M Sharifi H Mirzazadeh A Mehmandoost S Hosseini-Hooshyar S Ghalekhani N. A national study of suicidal ideation and suicide attempt among incarcerated people in Iran. Int J Ment Health Addict. (2023) 21:3043–60. doi: 10.1007/s11469-022-00773-6

[6] Lehoczki Á. Suicide and self-harm in prison [Öngyilkosság és önkárosítás a börtönvilágban]. Börtönügyi Szemle. (2012) 2012:33–40.

[7] Lehoczki Á. Analysis of completed suicides in the hungarian correctional system [a magyar büntetés-végrehajtásban történt befejezett szuicidumok elemzése]. Börtönügyi Szemle. (2015) 2015:64–75.

[8] Rutkai K Sánta L. Prisoners statistics: data related to incarceration [Fogvatartotti statisztikák: fogvatartással összefüggő Adatok]. Börtönstatisztikai Szemle. (2020) 2020:6–10.

[9] Castillejos MC Huertas P Martín P Moreno Küstner B. Prevalence of suicidality in the european general population: A systematic review and meta-analysis. Arch Suicide Res. (2021) 25:810–28. doi: 10.1080/13811118.2020.1765928 32620069

[10] Moore KE Siebert S Brown G Felton J Johnson JE. Stressful life events among incarcerated women and men: association with depression, loneliness, hopelessness, and suicidality. Health Justice. (2021) 9:22–. doi: 10.1186/s40352-021-00140-y PMC8386053 34427798

[11] Marzano L Hawton K Rivlin A Smith EN Piper M Fazel S. Prevention of suicidal behavior in prisons: an overview of initiatives based on a systematic review of research on near-lethal suicide attempts. Crisis. (2016) 37:323–34. doi: 10.1027/0227-5910/a000394 PMC5120691 27278569

[12] Wolff N Shi J. Childhood and adult trauma experiences of incarcerated persons and their relationship to adult behavioral health problems and treatment. Int J Environ Res Public Health. (2012) 9:1908–26. doi: 10.3390/ijerph9051908 PMC3386595 22754481

[13] Franke I Vogel T Eher R Dudeck M. Prison mental healthcare: recent developments and future challenges. Curr Opin Psychiatry. (2019) 32:342–7. doi: 10.1097/yco.0000000000000504 30855296

[14] Baranyi G Fazel S Langerfeldt SD Mundt AP. The prevalence of comorbid serious mental illnesses and substance use disorders in prison populations: A systematic review and meta-analysis. Lancet Public Health. (2022) 7:E557–E68. doi: 10.1016/S2468-2667(22)00093-7 PMC9178214 35660217

[15] Bedaso A Ayalew M Mekonnen N Duko B. Global estimates of the prevalence of depression among prisoners: A systematic review and meta-analysis. Depress Res Treat. (2020) 2020:3695209. doi: 10.1155/2020/3695209 33294222 PMC7718061

[16] Fazel S Seewald K. Severe mental illness in 33,588 prisoners worldwide: systematic review and meta-regression analysis. Br J Psychiatry. (2012) 200:364–73. doi: 10.1192/bjp.bp.111.096370 22550330

[17] van Buitenen N van den Berg CJW Meijers J Harte JM. The prevalence of mental disorders and patterns of comorbidity within a large sample of mentally ill prisoners: A network analysis. Eur Psychiatry. (2020) 63:e63–e. doi: 10.1192/j.eurpsy.2020.63 PMC7355171 32522312

[18] Fazel S Hayes AJ Bartellas K Clerici M Trestman R. Mental health of prisoners: prevalence, adverse outcomes, and interventions. Lancet Psychiatry. (2016) 3:871–81. doi: 10.1016/S2215-0366(16)30142-0 PMC5008459 27426440

[19] O'Connor RC Kirtley OJ. The integrated motivational-volitional model of suicidal behaviour. Philos Trans R Soc London Ser B Biol Sci. (2018) 373:1–10. doi: 10.1098/rstb.2017.0268 PMC6053985 30012735

[20] Hasking P Whitlock J Voon D Rose A. A cognitive-emotional model of nssi: using emotion regulation and cognitive processes to explain why people self-injure. Cogn Emotion. (2017) 31:1543–56. doi: 10.1080/02699931.2016.1241219 27702245

[21] Nock MK Prinstein MJ. A functional approach to the assessment of self-mutilative behavior. J consulting Clin Psychol. (2004) 72:885–90. doi: 10.1037/0022-006X.72.5.885 15482046

[22] Kresznerits S Zinner-Gérecz Á Perczel-Forintos D. [Borderline personality disorder and non-suicidal self-injury: the role of mindfulness training in risk reduction]. Psychiatr Hung. (2023) 38:142–52. 37439291

[23] Delgado-Gornez D Blasco-Fontecilla H Alegria AA Legido-Gil T Artes-Rodriguez A Baca-Garcia E. Improving the accuracy of suicide attempter classification. Artif Intell Med. (2011) 52:165–8. doi: 10.1016/j.artmed.2011.05.004 21696929

[24] Blasco-Fontecilla H Artieda-Urrutia P Berenguer-Elias N Garcia-Vega JM Fernandez-Rodriguez M Rodriguez-Lomas C. Are major repeater patients addicted to suicidal behavior? Adicciones. (2014) 26:321–33. doi: 10.20882/adicciones.26.4 25580865

[25] Blasco-Fontecilla H Fernández-Fernández R Colino L Fajardo L Perteguer-Barrio R de Leon J. The addictive model of self-harming (Non-suicidal and suicidal) behavior. Front Psychiatry. (2016) 7:8. doi: 10.3389/fpsyt.2016.00008 26869941 PMC4734209

[26] Nixon MK Cloutier PF Aggarwal S. Affect regulation and addictive aspects of repetitive self-injury in hospitalized adolescents. J Am Acad Child Adolesc Psychiatry. (2002) 41:1333–41. doi: 10.1097/00004583-200211000-00015 12410076

[27] Himelein-Wachowiak M Giorgi S Kwarteng A Schriefer D Smitterberg C Yadeta K. Getting “Clean” from nonsuicidal self-injury: experiences of addiction on the subreddit R/selfharm. J Behav Addict. (2022) 11:128–39. doi: 10.1556/2006.2022.00005 PMC9109623 35312631

[28] Guérin-Marion C Martin J Deneault A-A Lafontaine M-F Bureau J-F. The functions and addictive features of non-suicidal self-injury: A confirmatory factor analysis of the ottawa self-injury inventory in a university sample. Psychiatry Res. (2018) 264:316–21. doi: 10.1016/j.psychres.2018.04.019 29665561

[29] Goodman A. Addiction: definition and implications. Br J Addict. (1990) 85:1403–8. doi: 10.1111/j.1360-0443.1990.tb01620.x 2285834

[30] Washburn JJ Juzwin KR Styer DM Aldridge D. Measuring the urge to self-injure: preliminary data from a clinical sample. Psychiatry Res. (2010) 178:540–4. doi: 10.1016/j.psychres.2010.05.018 20580437

[31] Faye P. Addictive characteristics of the behavior of self-mutilation. J psychosocial Nurs Ment Health Serv. (1995) 33:36–9. doi: 10.3928/0279-3695-19950601-08 7666387

[32] Liu J Gao Y Liang C Liu X. The potential addictive mechanism involved in repetitive nonsuicidal self-injury: the roles of emotion dysregulation and impulsivity in adolescents. J Behav Addict. (2022) 11:953–62. doi: 10.1556/2006.2022.00077 PMC9881659 36287740

[33] Ying W Shen Y Ou J Chen H Jiang F Yang F. Identifying clinical risk factors correlated with addictive features of non-suicidal self-injury among a consecutive psychiatric outpatient sample of adolescents and young adults. Eur Arch Psychiatry Clin Neurosci. (2024) 274:291–300. doi: 10.1007/s00406-023-01636-4 37314538

[34] Viechtbauer W Smits L Kotz D Budé L Spigt M Serroyen J. A simple formula for the calculation of sample size in pilot studies. J Clin Epidemiol. (2015) 68:1375–9. doi: 10.1016/j.jclinepi.2015.04.014 26146089

[35] Garnefski N Kraaij V. The cognitive emotion regulation questionnaire: psychometric features and prospective relationships with depression and anxiety in adults. Eur J psychol Assess. (2007) 23:141–9. doi: 10.1027/1015-5759.23.3.141

[36] Geisler FCM Vennewald N Kubiak T Weber H. The impact of heart rate variability on subjective well-being is mediated by emotion regulation. Pers Individ Dif. (2010) 49:723–8. doi: 10.1016/j.paid.2010.06.015

[37] Miklósi M Martos T Kocsis-Bogár K Perczel Forintos D. A kognitív érzelem-reguláció Kérdőív magyar változatának pszichometriai jellemzôi. Psychiatria Hungarica. (2011) 26:102–11. 21653995

[38] Baer RA Smith GT Hopkins J Krietemeyer J Toney L. Using self-report assessment methods to explore facets of mindfulness. Assessment. (2006) 13:27–45. doi: 10.1177/1073191105283504 16443717

[39] Rózsa S Szádóczky E Füredi J. Psychometric properties of the hungarian version of the shortened beck depression inventory. Psychiatria Hungarica. (2001) 16:384–402.

[40] Beck AT Ward CH Mendelson M Mock J Erbaugh J. An inventory for measuring depression. Arch Gen Psychiatry. (1961) 4:561–71. doi: 10.1001/archpsyc.1961.01710120031004 13688369

[41] Cohen S Kamarck T Mermelstein R. A global measure of perceived stress. J Health Soc Behav. (1983) 24:385–96. doi: 10.2307/2136404 6668417

[42] Stauder A Konkoly Thege B. Az észlelt stressz kérdőíve (Pss) magyar verziójának jellemzői. Mentálhigiéné és Pszichoszomatika. (2006) 7:203–16. doi: 10.1556/Mental.7.2006.3.4

[43] Steinberg L Sharp C Stanford MS Tharp AT. New tricks for an old measure: the development of the barratt impulsiveness scale-brief (Bis-brief). psychol Assess. (2013) 25:216–26. doi: 10.1037/a0030550 23148649

[44] Horváthné Pató I Szekeres T Kresznerits S Perczel-Forintos D. [the barratt impulsiveness scale-brief-8 in an incarcerated sample: suicide risk, impulsivity and mindfulness]. Psychiatr Hung. (2023) 38:203–17. 37982268

[45] Paykel ES Myers JK Lindenthal JJ Tanner J. Suicidal feelings in the general population: A prevalence study. United Kingdom: R Coll Psychiatrists. (1974), 124:460–9. doi: 10.1192/bjp.124.5.460 4836376

[46] Nunnally JC Bernstein IH. Psychometric theory. 3rd Edition. New York: McGraw-Hill (1994).

[47] Fazel S Ramesh T Hawton K. Suicide in prisons: an international study of prevalence and contributory factors. Lancet Psychiatry. (2017) 4:946–52. doi: 10.1016/s2215-0366(17)30430-3 PMC6066090 29179937

[48] Kim HS Hodgins DC. Component model of addiction treatment: A pragmatic transdiagnostic treatment model of behavioral and substance addictions. Front Psychiatry. (2018) 9:406. doi: 10.3389/fpsyt.2018.00406 30233427 PMC6127248

[49] Ramirez-Castillo D Garcia-Roda C Guell F Fernandez-Montalvo J Bernacer J Morón I. Frustration tolerance and personality traits in patients with substance use disorders. Front Psychiatry. (2019) 10:421. doi: 10.3389/fpsyt.2019.00421 31258496 PMC6588127

[50] Janca E Keen C Willoughby M Borschmann R Sutherland G Kwon S. Sex differences in suicide, suicidal ideation, and self-harm after release from incarceration: A systematic review and meta-analysis. Soc Psychiatry Psychiatr Epidemiol. (2023) 58:355–71. doi: 10.1007/s00127-022-02390-z PMC9971066 36462041

